# Prevalence and impact of non-prescription medication misuse in the geriatric population

**DOI:** 10.1016/j.rcsop.2025.100663

**Published:** 2025-09-30

**Authors:** Moaddey Alfarhan, Jala Ashqar, Jawaher Ajeebi, Munira Ghazwani, Nouf Alnahdi, Yunus Yatimi, Talal AlMohammed, Khalid Khubrani, Dania Saleh, Haya Alsharif, Saeed A. Alqahtani

**Affiliations:** a Department of Clinical Practice, College of Pharmacy, Pharmacy Practice Research Unit, Jazan University, Jazan, Saudi Arabia; b College of Pharmacy, Pharmacy Practice Research Unit, Jazan University, Jazan, Saudi Arabia; c College of Pharmacy, King Saud University, Riyadh, Saudi Arabia; d College of Pharmacy, King Abdulaziz University, Jeddah, Saudi Arabia

**Keywords:** Over-the-counter medications, Geriatric patients, Medication misuse, Saudi Arabia, Drug-drug interactions, Health literacy, Patient education

## Abstract

**Background:**

Geriatric patients constitute the largest consumers of non-prescription medications. Understanding their patterns and consequences is essential.

**Aim:**

This study investigated OTC medication misuse among adults aged 65 and older in Saudi Arabia. It aimed to determine the extent of misuse, identify common medication classes, and analyze associated health risks.

**Methods:**

The study used a cross-sectional approach with 386 participants from various cities. Data were collected using a structured questionnaire delivered via two modes: an online survey and in-person interviews addressing demographics, medication use, drug interaction awareness, and educational needs. The sample ensured statistical accuracy, with a 5 % margin of error and a 95 % confidence level.

**Results:**

The majority of participants (80.7 %) used over-the-counter medications, primarily painkillers (66.4 %). Notably, 28.5 % use five or more regular medications. Some exhibited misuse behaviors, including overdosing (14.2 %) and using OTC medications for non-recommended purposes. Additionally, 24.9 % reported drug-drug interactions. Awareness gaps were significant: 39.4 % were unaware of the dangers of misuse, 38.3 % did not know potential side effects, 43.5 % were unaware of interactions with prescribed medications, and 56.2 % did not know about contraindications. Correlation analysis revealed that participants aware of potential side effects (80.1 %) were less likely to misuse OTC drugs than those unaware (89.2 %) (*p* = 0.046), and those who understood contraindications (75.5 %) showed lower misuse rates than the unaware (86.6 %) (*p* = 0.049).

**Conclusion:**

The study showed that most participants frequently used OTC medications, primarily analgesics, yet lacked knowledge about these drugs. It underscores the urgent need for interventions to prevent OTC misuse among the aging population, focusing on enhancing health literacy and safe drug practices. Recommended strategies include media campaigns and clinical programs to raise awareness about OTC misuse risks.

## 1 Introduction

The WHO defines the geriatric age group as older adults, who are individuals over the age of 65 years.^1^ By 2030, one in six individuals will be sixty or older, increasing the number of people in this age group from one billion in 2020 to 1.4 billion. The number of people in the world who are 60 years of age or older is expected to increase to 2.1 billion by 2050.^2^ On the local scale, by the end of 2050, the population in Saudi Arabia will reach 40 million, with older adults contributing about 25 % of the overall population.^1^

With this significant increase in the elderly population across the world, the prevalence of chronic and acute illnesses is also expected to increase as well. Additionally, the demand for medication is expected to rise, particularly for over-the-counter (OTC) medicines. Although OTC drugs reduce the number of visits to clinicians, decrease the cost, and save time, misuse of these drugs may result in side effects, drug-drug reactions, and overdosing, adversely affecting the individuals.^3^ OTC medicine is also known as non-prescription medication that does not require a prescription to use.^4^ Depending on the population and nation, the prevalence of using OTC medications varies significantly throughout the world, ranging from 11.2 % to 93.7 %.^5^^,^^6^

In Saudi Arabia, citizens benefit from free healthcare services provided by the government, which grants access to both prescription medications and certain over-the-counter (OTC) drugs within public healthcare facilities.^7^ Meanwhile, OTC products are readily available in community pharmacies and are usually purchased out-of-pocket. Despite the existence of several regulations governing OTC consumption in the country, approximately 81 % of the general population has reported using OTC medications at some point in their lives. However, this rise in usage has not been correlated with improved health outcomes. According to the World Health Organization (WHO), nearly 50 % of patients misuse these drugs, leading to increased morbidity and mortality rates.^8^ The inappropriate use of OTC medications can also result in significant drug-drug interactions and a rise in healthcare utilization, including hospitalizations.^9^

Misuse of OTC medications occurs when individuals take the medication in a way or dosage other than directed on the package or administer them for the effect it produces, either to get high or mix OTC medicines to create new products.^10^ Because OTCs do not require a prescription, there is limited knowledge or consideration of how these medications are used, including the extent of their misuse.^11^ Results indicate that 80 % of older adults using OTC products demonstrated probable misuse in the United States.^12^ Older adults are especially susceptible to adverse drug events (ADEs) due to various age-related factors, including changes in pharmacokinetics and pharmacodynamics, multimorbidity, polypharmacy, health condition complexity, declining cognition, and general frailty.^13^

This study is relevant to clinical and social pharmacy, as clinical pharmacists educate patients, identify inappropriate medication use, and conduct medication reconciliation, particularly among the elderly. From a social pharmacy standpoint, understanding beliefs, behaviors, and knowledge affecting OTC use is key for public health interventions and policies to improve medication safety. By identifying misuse patterns and knowledge gaps, it supports pharmacy-led community education and highlights the need for policy support for medication counseling. The study investigated the impact of OTC drug misuse on older patients' health, including prevalence, types of misuse, and related health issues. It also examined factors such as polypharmacy and explored negative outcomes, including hospitalization. The results will improve awareness and management of OTC use among the elderly, guiding healthcare practices and policies in Saudi Arabia.

## 2 Methodology

### 2.1 Study design and setting

This cross-sectional study, conducted from November 2024 to March 2025 across multiple Saudi cities, including Riyadh, Jeddah, and Jizan, targeted adults aged 65 and older. Data were collected from the elderly or their caregivers to capture their perspectives.

### 2.2 Questionnaire development

A structured questionnaire was developed based on an extensive review of existing literature on OTC medication use and misuse among elderly populations, incorporating insights from studies in both Saudi Arabia and international contexts. The final questionnaire consisted of six domains:

1. Demographic and socioeconomic characteristics: age, gender, marital status, educational level, employment status, monthly income, nationality, and living arrangement.
2. Health-related information: presence and type of chronic diseases, use of prescription medications, and total number of medications (prescription + OTC).
3. Patterns of OTC medication use: types of medications used, frequency, and reasons for use.
4. Misuse behaviors: including overdose, use for non-recommended purposes, and combining OTC with prescription medications.
5. Knowledge and awareness – understanding of side effects, drug–drug interactions, contraindications, and safe use.
6. Educational needs and preferred educational methods.

### 2.3 Questionnaire creation process

The process involved creating questions based on a review of existing literature on OTC medication use and misuse among the elderly, incorporating global and Saudi studies. To ensure the instrument's content validity, two experts in clinical pharmacy conducted a thorough review of the draft questionnaire, assessing its content validity, cultural appropriateness, and comprehensiveness. The revised questionnaire was piloted with 20 elderly participants to evaluate clarity, language suitability, and completion time.

### 2.4 Inclusion and exclusion criteria

Adults aged 65 years and older living in Saudi Arabia at the time of the study were eligible to participate if they had used OTC medications at least once in the past 12 months and were able to provide informed consent, either on their own or with help from a caregiver. Exclusion criteria included individuals younger than 65 years or those who had never used OTC medications.

### 2.5 Sample size calculation

Sample size was calculated using the Raosoft® online calculator, assuming a 50 % response distribution, a 95 % confidence level, and a 5 % margin of error. The required minimum sample size was 384 participants, and the study successfully recruited 386.

### 2.6 Data collection procedure

The questionnaire was administered online using Google Forms. For participants with limited digital literacy but with caregiver support, responses were recorded by the caregiver under the participant's direction. Recruitment materials included a study description and eligibility criteria, and informed consent was obtained electronically before participation.

### 2.7 Data analysis

Data were analyzed using IBM SPSS Statistics Version 29 (IBM Corp., Armonk, NY). Descriptive statistics (frequencies, percentages, and standard deviations) were used to summarize participant characteristics and OTC usage patterns. Associations between OTC misuse and variables such as age, sex, comorbidities, and awareness of interactions were assessed using chi-square tests. The significance level of *p* < 0.05 was considered statistically significant.

### 2.8 Ethical considerations

Ethical approval for this study was obtained from the Jazan University Institutional Review Board (IRB Approval No. REC-46/06/1307). Participation was voluntary, and informed electronic consent was obtained from all respondents prior to starting the survey. Data confidentiality was ensured by anonymizing responses and storing them in password-protected files accessible only to the research team.

## 3 Results

### 3.1 Characteristics of study

A total of 386 geriatric patients were included in this study. A significant majority of patients fell within the 65–69 age group (*N* = 248, 64.2 %) (Table 1). Females comprised a larger portion of the sample (*N* = 221, 57.3 %) than males (*N* = 165, 42.7 %). Regarding marital status, most participants were married (*N* = 242, 62.7 %), while 26.9 % were divorced or widowed (*N* = 104). Educational levels varied, with 34.5 % of the participants being illiterate (*N* = 133). In terms of employment, a considerable portion were retired (*N* = 170, 44.0 %) (Table 1). The monthly income distribution showed that 34.2 % earned less than 3000 Saudi Riyals (SR) (*N* = 132). Most participants were Saudi nationals (*N* = 364, 94.3 %) and lived with their family or friends (*N* = 349, 90.4 %).

**Table 1:** Demographic and Health-Related Variables of Geriatric Patients (*N* = 386).

Variables	No	%
**Age in years**		
65–69	248	64.2 %
70–74	84	21.8 %
75–80	29	7.5 %
> 80	25	6.5 %
**Gender**		
Male	165	42.7 %
Female	221	57.3 %
**Marital status**		
Single	40	10.4 %
Married	242	62.7 %
Divorced / widow	104	26.9 %
**Educational level**		
Illiterate	133	34.5 %
Secondary / below	115	29.8 %
University graduate	114	29.5 %
Post-graduate	24	6.2 %
**Employment**		
Unemployed	162	42.0 %
Employee	54	14.0 %
Retired	170	44.0 %
**Monthly income**		
< 3000 SR	132	34.2 %
3000–5999 SR	109	28.2 %
6000–9999 SR	65	16.8 %
10,000–20,000 SR	61	15.8 %
> 20,000 SR	19	4.9 %
**Nationality**		
Saudi	364	94.3 %
Non-Saudi	22	5.7 %
**Living with whom?**		
Live with family/friends	349	90.4 %
Live alone	37	9.6 %

A substantial proportion reported having chronic diseases, with diabetes mellitus (DM) being the most prevalent (N = 192, 49.7 %), followed by hypertension (HTN) (*N* = 184, 47.7 %) (Table 2). Notably, 84.7 % of the participants reported using prescription medications (*N* = 327), with 28.5 % taking five or more medications (N = 110) (Table 2).

**Table 2:** Health-Related Variables of Geriatric Patients (*N* = 386).

Variables	No	%
**Have chronic diseases**		
None	93	24.1 %
Diabetes Mellitus	192	49.7 %
Hypertension	184	47.7 %
Cardiac diseases	101	26.2 %
Hepatic diseases	95	24.6 %
Bone/osteoporosis	25	6.5 %
Renal diseases	15	3.9 %
Others	17	4.4 %
**Do you use prescription medications?**		
Yes	327	84.7 %
No	59	15.3 %
**Total number of drugs received (including prescribed and OTC)**		
None	39	10.1 %
1 drug	31	8.0 %
2 drugs	82	21.2 %
3 drugs	67	17.4 %
4 drugs	57	14.8 %
≥ 5 drugs	110	28.5 %

### 3.2 The distribution of OTC medication usage among geriatric patients

A significant majority reported using OTC medications (*N* = 312, 80.7 %). Among those who used OTC medications, analgesics were the most reported, with 255 participants (66.4 %) indicating their use. Following analgesics, antispasmodic medications (*N* = 110, 28.6 %), vitamins (*N* = 120, 31.3 %), and antacids (N = 105, 27.3 %) were the most frequently used medications. Anti-allergic medications were reported by 77 participants (20.1 %), while herbs and herb-containing medications were used by 73 participants (19.0 %). Sleep aids were used by 61 participants (16.1 %), and a small number reported using other OTC medications (*N* = 2, 0.5 %). Conversely, 62 participants (19.3 %) reported never using OTC medications.

### 3.3 Assessment of OTC medication use behaviors and related factors in geriatric patients

Regarding the reasons for OTC medication use, 45.1 % (*N* = 173) cited easy access, while 42.4 % (*N* = 163) stated a preference against their use, and 32.0 % (*N* = 123) reported using OTCs to avoid doctors' visits. In terms of misuse, a total of 14.2 % (*N* = 55) reported taking an OTC overdose; furthermore, 33.2 % (*N* = 128) reported using OTCs for purposes other than those recommended. Regarding medication interactions, a notable 49.0 % of participants (*N* = 189) reported that they occasionally combined prescription medications with OTC medications. However, only 37.6 % (*N* = 145) actively maintained comprehensive medication records, leaving the majority, 62.4 % (*N* = 241), without such documentation. Among those surveyed, 24.9 % (*N* = 96) experienced side effects after taking multiple medications, with an additional 24.4 % (*N* = 94) expressing uncertainty about potential adverse reactions. Awareness among doctors about their patients' other medications varied significantly; 43.5 % (*N* = 168) indicated that they sometimes discussed their patients' medication regimens, while 27.2 % (*N* = 105) consistently engaged in these conversations. Similarly, discussions about medication interactions with healthcare providers occurred occasionally for 37.8 % (*N* = 146) of the respondents. Most respondents (53.4 %, *N* = 206) mentioned that healthcare professionals had advised against specific OTCs due to health conditions. Finally, 49.5 % (*N* = 191) were responsible for their own medication administration, while 42.5 % (*N* = 164) relied on family or friends.

### 3.4 Geriatric patients' knowledge and awareness regarding non-prescription medication use

Among the respondents, 57.8 % (*N* = 223) indicated that they had received information on the safe use of medications, while a significant number, 42.2 % (*N* = 163), reported that they had not. Additionally, 60.6 % (*N* = 234) were aware of the dangers associated with misuse; however, 39.4 % (*N* = 152) lacked this knowledge. The awareness of interactions between OTC and prescription medications was particularly low, with only 34.7 % (*N* = 134) acknowledging such interactions. Meanwhile, 43.5 % (*N* = 168) were unaware, and 21.8 % (*N* = 84) were unsure about these interactions. Regarding the effects of OTC medications on chronic conditions, 53.9 % (*N* = 208) were informed, while a notable portion, 27.2 % (*N* = 105), were not, and 18.9 % (*N* = 73) expressed uncertainty. Awareness of potential side effects was also limited, with 36.5 % (*N* = 141) being aware, 38.3 % (*N* = 148) being unaware, and 25.1 % (*N* = 97) being unsure. Lastly, a majority of respondents, 56.2 % (*N* = 217), were unaware of the contraindications associated with OTC medications, whereas 43.8 % (*N* = 169) stated that they were aware of them.

### 3.5 Practices, perceptions, and educational needs of geriatric patients regarding OTC medication use

The study assessed the practices, perceptions, and educational needs of geriatric patients regarding the use of OTC medications. Among participants with chronic conditions, 30.1 % (*N* = 116) reported that they consistently considered these conditions when taking OTCs, while 15.8 % (*N* = 61) stated they never did so. Regarding reading package instructions, 28.5 % (*N* = 110) stated that they always read them, while 20.5 % (*N* = 79) admitted to never reading the instructions. Consultation with a pharmacist varied, with 38.3 % (N = 148) always seeking advice, while 8.0 % (*N* = 31) never did. A significant majority, 85.8 % (*N* = 331), believed it is essential for healthcare professionals to inquire about OTC use, and 87.0 % (*N* = 336) recognized the need for greater awareness about OTC misuse, especially among the elderly. Furthermore, 79.8 % (*N* = 308) expressed interest in receiving educational materials on the safe use of non-prescription medications.

### 3.6 Suggested methods for raising awareness about OTC medication misuse in geriatric patients

Television and radio campaigns emerged as the most preferred method, with 50.5 % (*N* = 195) of participants advocating this approach. Following closely were educational programs in healthcare centers, which were favored by 49.7 % (*N* = 192) of respondents. SMS notifications were deemed effective by 46.9 % (*N* = 181), while posts from pharmacies garnered support from 45.1 % (*N* = 174). Additionally, social media was recommended by 44.6 % (*N* = 172) of participants. In stark contrast, emails were the least favored option, with only 15.3 % (*N* = 59) suggesting them. Lastly, 7.8 % (N = 30) proposed alternative methods.

### 3.7 Factors associated with elderly OTC medications misuse

A lower percentage of single individuals (65.0 %) reported using OTC medications compared to married (84.7 %) and divorced/widowed individuals (89.4 %) (*p* = 0.001). Similarly, a higher percentage of unemployed individuals (92.0 %) reported using OTCs compared to employees (79.6 %) and retired individuals (77.6 %) (p = 0.001). Participants with chronic diseases showed a higher percentage of OTC use (86.2 %) compared to those without chronic diseases (76.1 %) (*p* = 0.023). Finally, when considering chronic conditions during OTC use, a higher percentage of those who sometimes took them into account used OTCs (89.9 %) compared to those who never did (77.0 %) or always did (80.2 %) (*p* = 0.048) (Table 3).

**Table 3:** Factors associated with elderly OTC medications misuse.

Factors		Used OTC medications				*p*-value
		Yes		No		
		No.	%	No.	%	
**Age in years**	65–69	209	84.3 %	39	15.7 %	0.590^
	70–74	70	83.3 %	14	16.7 %	
	75–80	26	89.7 %	3	10.3 %	
	> 80	19	76.0 %	6	24.0 %	
**Gender**	Male	135	81.8 %	30	18.2 %	0.327
	Female	189	85.5 %	32	14.5 %	
**Marital status**	Single	26	65.0 %	14	35.0 %	0.001*
	Married	205	84.7 %	37	15.3 %	
	Divorced / widow	93	89.4 %	11	10.6 %	
**Educational level**	Illiterate	119	89.5 %	14	10.5 %	0.122
	Secondary / below	92	80.0 %	23	20.0 %	
	University graduate	95	83.3 %	19	16.7 %	
	Post-graduate	18	75.0 %	6	25.0 %	
**Employment**	Unemployed	149	92.0 %	13	8.0 %	0.001*
	Employee	43	79.6 %	11	20.4 %	
	Retired	132	77.6 %	38	22.4 %	
**Monthly income**	< 3000 SR	115	87.1 %	17	12.9 %	0.638
	3000–5999 SR	92	84.4 %	17	15.6 %	
	6000–9999 SR	53	81.5 %	12	18.5 %	
	10,000–20,000 SR	48	78.7 %	13	21.3 %	
	> 20,000 SR	16	84.2 %	3	15.8 %	
**Nationality**	Saudi	304	83.5 %	60	16.5 %	0.359^
	Non-Saudi	20	90.9 %	2	9.1 %	
**Living with whom?**	Live with family/friends	296	84.8 %	53	15.2 %	0.150
	Live alone	28	75.7 %	9	24.3 %	
**Have chronic diseases**	Yes	257	86.2 %	41	13.8 %	0.023*
	No	67	76.1 %	21	23.9 %	
**Discussed interactions or interactions that may occur with your medications with your healthcare provider, such as doctors or pharmacists?**	Never	66	79.5 %	17	20.5 %	0.235
	Rarely	51	85.0 %	9	15.0 %	
	Sometimes	129	88.4 %	17	11.6 %	
	Always	78	80.4 %	19	19.6 %	
**Have you ever been advised by a healthcare professional not to take any specific non-prescription medication because of your health condition?**	Yes	175	85.0 %	31	15.0 %	0.562
	No	149	82.8 %	31	17.2 %	
	No	46	83.6 %	9	16.4 %	


Factors		Used OTC medications				p-value
		Yes		No		
		No.	%	No.	%	
**Have you taken your chronic conditions into account when taking over-the-counter medications?**	Never	47	77.0 %	14	23.0 %	0.048*
	Rarely	42	82.4 %	9	17.6 %	
	Sometimes	142	89.9 %	16	10.1 %	
	Always	93	80.2 %	23	19.8 %	
**Do you think healthcare professionals should ask about your use of over-the-counter medications?**	Yes	278	84.0 %	53	16.0 %	0.948
	No	46	83.6 %	9	16.4 %	

Table 3 presents the factors associated with the misuse of over-the-counter (OTC) medications among the elderly, based on a sample size of 386 individuals. The analysis was conducted using the Pearson Chi-square test (P), with an exact probability test denoted by the symbol (^). Results with a *p*-value ≤0.05 (*) are considered statistically significant.

### 3.8 The correlation between awareness levels and OTC medication misuse

The analysis in Table 4 establishes a direct correlation between awareness levels and OTC medication misuse. Participants who were aware of potential side effects (80.1 %) were significantly less likely to misuse OTC drugs compared to those unaware (89.2 %) (*p* = 0.046). In comparison, those who understood contraindications (75.5 %) exhibited lower misuse rates than those unaware (86.6 %) (*p* = 0.049). These findings align with Table 4, which shows that a significant percentage of participants, 56.2 % were unaware of contraindications, reinforcing the notion that inadequate knowledge contributes to improper medication use. However, broader awareness of general medication risks, such as safe usage guidelines, misuse dangers, drug interactions, and the impact on chronic conditions, did not show significant associations with OTC use (*p* > 0.05).

**Table 4:** Association between elderly awareness about OTC medications and their misuse.

Knowledge	Used OTC medications				p-value
	Yes		No		
	No.	%	No.	%	
**Have you ever received any information or instructions about the safe use of non-prescription medications?**					0.740
Yes	186	83.4 %	37	16.6 %	
No	138	84.7 %	25	15.3 %	
**Do you know the dangers and complications of misusing non-prescription drugs?**					0.906
Yes	196	83.8 %	38	16.2 %	
No	128	84.2 %	24	15.8 %	
**Are you aware of potential interactions between over-the-counter and prescription medications?**					0.765
Yes	110	82.1 %	24	17.9 %	
No	143	85.1 %	25	14.9 %	
Not sure	71	84.5 %	13	15.5 %	
**Did you know that some over-the-counter medications can negatively affect chronic health conditions?**					0.317
Yes	171	82.2 %	37	17.8 %	
No	93	88.6 %	12	11.4 %	
Not sure	60	82.2 %	13	17.8 %	
**Are you aware of the potential side effects of the over-the-counter medications you are taking?**					0.046*
Yes	113	80.1 %	28	19.9 %	
No	132	89.2 %	16	10.8 %	
Not sure	79	81.4 %	18	18.6 %	
**Do you know the contraindications of over-the-counter medications?**					0.049*
Yes	136	80.5 %	33	19.5 %	
No	188	86.6 %	29	13.4 %	

Table 4 illustrates the association between elderly awareness of over-the-counter (OTC) medication and their misuse, evaluated using the Pearson Chi-Square test. Results with a p-value ≤0.05 (*) are considered statistically significant.

## 4 Discussion

Aging is a gradual and irreversible biological process that involves the decline in the function of tissues and cells. This decline significantly increases the likelihood of developing various age-related diseases, such as neurodegenerative, cardiovascular, metabolic, musculoskeletal, and immune disorders.^14^ The rise in morbidity contributes to the development of extensive geriatric syndromes.^15^^,^^16^ Multimorbidity is associated with a wide range of polypharmacy commonly prescribed by several healthcare providers.^16^ Additionally, elderly individuals often purchase OTC medications and dietary supplements independently.^17^ OTC drugs are widely available, allowing individuals to self-treat common illnesses without a prescription or the cost of seeing a doctor. While these medications are generally considered safe, there is still a risk of adverse health effects.^11^

This study examined the patterns, knowledge, practices, and factors related to OTC medication misuse among elderly patients in Saudi Arabia. The findings offer critical insights into the sociodemographic and health-related factors that contribute to OTC misuse, as well as gaps in awareness and educational needs. These results both align with and differ from previous research, highlighting universal and context-specific challenges in geriatric self-medication. In this study, 34.5 % of participants were illiterate, which could significantly affect health literacy; additionally, 89.5 % of illiterate participants were using OTC medications. As emphasized by Rabia Shahid (2022), low health literacy is associated with poorer health outcomes and a higher risk of medication errors.^18^ Illiteracy may hinder understanding of medication labels, instructions, and potential side effects, thereby increasing the risk of misuse. This highlights the importance of targeted educational efforts aimed at improving health literacy in this population.

Regarding medication usage patterns, a significant portion (80.7 %) of participants reported using OTC medications, with analgesics being the most used class, as 66.4 % of participants indicated their use. This finding supports the research by Huang YL (2020), which identified pain management as a key reason for OTC medication use among older adults.^14^ The high prevalence of chronic diseases, particularly diabetes (49.7 %) and hypertension (47.7 %), mirrors the findings from the World Health Organization, which states that older adults frequently manage multiple chronic conditions, leading to an increase in medication use.^19^ The relationship between chronic conditions and OTC usage suggests that healthcare providers should consider these factors when advising patients on medications. However, the frequent use of antispasmodic medications (28.6 %) and antacids (27.3 %) in this group may reflect regional dietary habits, such as high consumption of spicy foods or traditional remedies, which are linked to gastrointestinal problems in Saudi Arabia.^20^

The prevalence of polypharmacy among a nationally representative cohort of older adults in Saudi Arabia was notably high, at 51.5 %.^21^ These results showed that 28.5 % of participants were taking five or more medications, raising concerns about polypharmacy. Kouladjian et al. (2020) found that polypharmacy is common among older adults and often leads to complications due to drug interactions.^22^ This study reinforces the idea that as elderly patients deal with multiple health issues, the risk of adverse drug events rises, requiring careful medication management and regular reviews by healthcare providers.

Although 57.8 % of participants reported having received information about the safe use of OTC medications, a considerable number remained unaware of potential side effects (38.3 %) and contraindications (56.2 %). This lack of awareness is concerning, as inadequate knowledge of medication risks can lead to misuse. The significance of proper counseling for older adults regarding the risks of self-medication is emphasized by Shideh et al. (2023), who underscored the critical role of healthcare providers in delivering comprehensive medication education.^23^ The top strategies preferred for raising awareness among participants included TV and radio campaigns (50.5 %) and programs at healthcare centers (49.7 %). This study indicates a strong preference among elderly individuals in Saudi Arabia for traditional, face-to-face communication methods. The popularity of television, radio, and healthcare center programs underlines the importance of utilizing familiar and trusted channels when developing awareness campaigns aimed at this age group. While SMS and pharmacy-based messages received moderate acceptance, the limited preference for email and social media suggests that digital strategies should be approached with caution and complemented by more accessible methods. These findings highlight the necessity of age-appropriate and culturally relevant communication to engage the elderly population effectively.

The limited understanding of interactions between OTC and prescription medications (34.7 %) is particularly troubling. According to Shideh et al. (2023), older adults face a higher risk of adverse drug events when they do not fully comprehend how different medications might interact.^23^ This finding demonstrates the necessity for healthcare providers to engage in thorough medication reconciliation and counseling to ensure patients are aware of the potential consequences of combining medications.

This study revealed that 49.0 % of participants engaged in practices indicative of OTC medication misuse, such as using medications without consulting healthcare providers. This pattern aligns with the findings of Shideh et al. (2023), who identified similar self-medication trends in older populations.^23^ Many older adults may avoid seeking professional advice due to perceived barriers, such as accessibility issues or a lack of trust in healthcare providers. This situation highlights the importance of creating healthcare environments where older adults feel comfortable discussing their medication use.

Additionally, marital and employment status were identified as significant predictors of misuse. Unmarried individuals reported lower usage rates at 65.0 %, compared to 84.7 % among married individuals and 89.4 % among divorced or widowed individuals. These findings contrast with those of other studies, which indicate that individuals who are unmarried or lack social support are more likely to use OTC medications.^24^, ^25^, 26.

The study examined the attitudes and educational needs of the elderly regarding OTC medication use. Key findings include a significant majority (85.8 %) believe healthcare professionals should routinely ask about OTC medication use, reflecting a desire for comprehensive medication reviews. 87 % of participants recognize the need for increased education about the misuse of non-prescription medications, highlighting concerns about drug interactions and polypharmacy. 79.8 % express interest in learning about the safe use of OTC medications, indicating an opportunity for healthcare providers to engage with this population. There is a correlation between awareness of side effects and contraindications and lower rates of OTC drug misuse, suggesting that knowledge can protect against misuse. In addition, a notable portion of study participants (62.4 %) reported not having medication records, while only 37.6 % maintained such records. This highlights systemic shortcomings in medication management. The findings suggest that generic health warnings may not be adequate to influence patient behavior. This is further supported by the study's discovery that 49.0 % of participants occasionally combined prescription and OTC medications, thereby posing potential risks for adverse drug interactions. This study advocates for integrating routine inquiries about OTC use into medical practice and developing educational interventions tailored to older adults' needs to enhance medication safety and informed decision-making.

These findings may also be considered in light of the Health Belief Model (HBM), which explains health behaviors through perceived susceptibility, severity, benefits, and barriers.^27^ In the context of OTC misuse, older adults may underestimate their risk of drug interactions, downplay potential severity, and perceive self-medication as more convenient than professional care.^28^ Barriers such as limited health literacy and restricted access to counseling may further contribute. While the present study did not apply this framework, future research could use the HBM to better explain misuse patterns and inform targeted interventions.

## 5 Limitations

This study has several limitations that warrant consideration. First, the data were self-reported, which may have introduced recall bias and social desirability bias, as participants may have underreported the use of inappropriate medications or failed to recall all OTC and prescription medications accurately. Second, as the survey was administered online using Google Forms, participation was limited to elderly individuals with internet access and basic digital literacy, potentially excluding a subset of the older population, particularly those less familiar with technology, and limiting the representativeness of the sample. Third, participants with moderate to severe cognitive impairment were unlikely to participate or may have struggled to accurately complete the questionnaire, despite being a group at high risk for medication misuse and adverse interactions. Additionally, although the study was conducted mostly across three cities in Saudi Arabia to enhance geographic diversity, the findings may not be generalizable to all regions of the country, particularly rural areas, or to elderly populations in different cultural or healthcare settings.

Furthermore, despite efforts to simplify the questionnaire, there remains the possibility that some participants misinterpreted medical terminology or concepts, especially among those with lower health literacy levels. The cross-sectional nature of the study also limits the ability to draw causal inferences between OTC medication use and reported outcomes. Lastly, certain types of non-prescription products, such as herbal supplements and traditional remedies, may not have been consistently reported by participants if they did not perceive these as OTC medications, potentially leading to an underestimation of overall OTC use.

One limitation is that the response options for assessing chronic conditions (“never,” “rarely,” “sometimes,” “always”) were not tied to specific numerical thresholds. This absence of predetermined cut-offs might diminish the precision and comparability of responses. Future research should implement standardized quantitative measures to improve clarity.

A limitation of this study is that the analysis was primarily univariate, relying on chi-square tests to explore associations. This approach does not adjust for potential confounders such as comorbidities, education, or income, which may have influenced the observed relationships. Future studies with larger sample sizes should employ multivariate techniques, such as logistic regression, to control for these factors and provide a more robust understanding of predictors of OTC misuse.

## 6 Conclusion

This study emphasizes the need for urgent interventions to address OTC medication misuse among older adults. Misuse, amid knowledge gaps, highlights the importance of improving health literacy and safe drug use. Findings can guide future prevention efforts, with education campaigns on TV, radio, and clinical programs playing key roles in raising awareness of OTC risks. Long-term studies are necessary to assess the impact of education and technology on medication adherence and safety.

## Declaration of generative AI in scientific writing

During the preparation of this work, the authors used ChatGPT and Grammarly in order to enhance the manuscript's readability and language. After using this tool/service, the author(s) reviewed and edited the content as needed and take full responsibility for the content of the published article.

## CRediT authorship contribution statement

**Moaddey Alfarhan:** Writing – review & editing, Validation, Supervision, Project administration, Methodology, Investigation, Funding acquisition, Conceptualization. **Jala Ashqar:** Writing – original draft, Methodology, Investigation, Data curation, Conceptualization. **Jawaher Ajeebi:** Writing – original draft, Methodology, Investigation, Data curation, Conceptualization. **Munira Ghazwani:** Writing – original draft, Methodology, Investigation, Data curation, Conceptualization. **Nouf Alnahdi:** Writing – original draft, Methodology, Investigation, Data curation, Conceptualization. **Yunus Yatimi:** Writing – original draft, Methodology, Investigation, Data curation, Conceptualization. **Talal AlMohammed:** Writing – original draft, Methodology, Investigation, Data curation. **Khalid Khubrani:** Writing – original draft, Methodology, Investigation, Data curation, Conceptualization. **Dania Saleh:** Writing – original draft, Methodology, Investigation, Data curation, Conceptualization. **Haya Alsharif:** Writing – original draft, Project administration, Methodology, Data curation, Conceptualization. **Saeed A. Alqahtani:** Writing – review & editing, Validation, Supervision, Project administration, Methodology, Investigation, Data curation, Conceptualization.

## Declaration of competing interest

The authors declare that they have no known competing financial interests or personal relationships that could have appeared to influence the work reported in this paper.

## Appendix A Supplementary data

Supplementary material 1 Image 1

Supplementary material 2 Image 2
