# Supplementary material for: Prevalence and impact of non-prescription medication misuse in the geriatric population
Source: Explor Res Clin Soc Pharm. 2025 Sep 30;20:100663. doi: 10.1016/j.rcsop.2025.100663 (PMC12547873; doi:10.1016/j.rcsop.2025.100663)
Supplement: Supplementary file 1 — Supplementary material 1 [file mmc1.docx]

**Supplementary File 1**: **Full questionnaire**

| **Section 1: Demographic Data** | |
| --- | --- |
| What is your age? | - 65–69 - 70–74 - 75–79 - 80 and above |
| What is your gender? | - Male - Female |
| What is your current marital status? | - Single - Married - Widowed - Divorced |
| What is your education level? | - No formal education - High school or less - Bachelor’s degree - Postgraduate degree |
| What is your employment status? | - Employed - Retired - Unemployed |
| What is your monthly income? | - Less than 3,000 SAR - 3,000–5,999 SAR - 6,000–9,999 SAR - 10,000-19,999 - 20,000 SAR or more |
| What is your citizenship? | - Saudi - Non-Saudi |
| Do you currently live alone or with others? | - I live alone - I live with others (e.g., family, friends) |
| **Section 2: Over-the-Counter (OTC) Medication Use** | |
| Do you use over-the-counter (OTC) medications? | - Yes - No |
| Why do you prefer using OTC medications? (Select all that apply) | - Easy to access - Avoid visiting the doctor - Believe they are safe - Other: |
| Which of these OTC medications do you use? (Select all that apply) | - Analgesics - Allergy medications - Antacids - Herbs or extracts - Multivitamins - Sleep aids - I do not use over-the-counter medications - Other:___ |
| Have you experienced any side effects from OTC medications? | - Yes - No |
| If yes, what side effects have you experienced? (Select all that apply) | - Nausea - Dizziness - Confusion - Drowsiness - Fall - Abdominal cramping or Stomach pain - Headache - Stomach ulcers - Bleeding - Kidney problems - Dry mouth - Bone fractures - Vitamin deficiencies - Dehydration - Electrolyte imbalance - Raise blood pressure - Increase in heart rate - Hypotension - Bleeding - Other: _______ |
| **Section 3: Prescription Medications** | |
| **Do you have any chronic health conditions? Please indicate if you have been diagnosed with any of the following conditions:** | - Diabetes (Type 1 or Type 2) - Hypertension (High Blood Pressure) - Heart Disease (e.g., Coronary Artery Disease, Heart Failure) - Kidney Disease (e.g., Chronic Kidney Disease) - Liver Disease (e.g., Hepatitis, Cirrhosis) - Arthritis (e.g., Osteoarthritis, Rheumatoid Arthritis) - Other: ___________ - **No, I do not have any chronic health conditions.** |
| Do you take prescription medications? | - Yes - No |
| What types of prescription medications do you take most frequently? (Select all that apply) | - Antihypertensives (blood pressure medications) - Antidiabetics - Cholesterol-lowering medications - Anticoagulants (blood thinners) - Antipsychotics - Antidepressants - Anti-anxiety - Pain relievers - Other: _______ |
| **Section 4: Assessment of OTC Medications Misuse** | |
| Have you ever taken a higher dose or more frequently of an OTC medication than recommended? | - Yes - No |
| Have you ever used an OTC medication for a purpose other than what it was intended for? | - Yes - No |
| Are you aware of the risks associated with the misuse of OTC medications? | - Yes - No |
| **Section 5: Assessment and Awareness of Drug Interactions** | |
| How many medications do you take regularly (including prescribed and OTC)? | - 1 - 2 - 3 - 4 - More than 4 |
| How often do you take prescription medications along with OTC medications? | - Always - Sometimes - Rarely - Never |
| Are you aware of potential interactions between OTC medications and prescription drugs? | - Yes - No - Not sure |
| Have you ever experienced side effects after taking more than one medication (whether prescription or OTC)? | - Yes - No - Not sure |
| Do you inform your doctor about the other prescribed and OTC medications you use? | - Always - Sometimes - Rarely - Never |
| Do you discuss the possible interactions of your medications with healthcare providers such as doctors or pharmacists? | - Always - Sometimes - Rarely - Never |
| **Section 6: Drug-Disease Interactions** | |
| Are you aware that some OTC medications can negatively affect chronic health conditions? | - Yes - No - Not sure |
| Do you consider your chronic conditions when choosing OTC medications? | - Always - Sometimes - Rarely - Never |
| Has your health condition ever worsened after taking an OTC medication? | - Yes - No - Not sure |
| **Section 7: Medication Practices and Safety** | |
| Do you keep a record of all the medications you are using (both prescription and OTC)? | - Yes - No |
| Do you read the instructions on OTC medication packaging? | - Always - Sometimes - Rarely - Never |
| Do you understand the possible side effects of the OTC medications you use? | - Yes - No - Not sure |
| Do you know the contraindications of OTC medications? | - Yes - No |
| Do you consult a pharmacist when purchasing OTC medications? | - Always - Sometimes - Rarely - Never |
| Who is primarily responsible for managing or administering your medications? | - I manage my medications myself - A family member or friend manages my medications - A healthcare provider (e.g., nurse, caregiver) manages my medications - Other: _______ |
| **Section 8: Educational Interventions** | |
| Do you believe that healthcare professionals should ask about your use of OTC medications? | - Yes - No |
| Have you ever been advised by a healthcare professional not to take a certain OTC medication due to your health condition? | - Yes - No |
| Have you ever received information or education about the safe use of OTC medications? | - Yes - No |
| Do you believe there is a need for more awareness about the misuse of OTC medications among the elderly? | - Yes - No |
| Would you be interested in receiving educational materials on the safe use of OTC medications? | - Yes - No |
| What do you think is the best way to raise awareness about the risks of OTC medication misuse? (Select all that apply) | - TV and radio campaigns - Educational programs at healthcare centers - Leaflets from pharmacies - SMS notifications - Emails - Social media |
