# Supplementary material for: Prevalence and impact of non-prescription medication misuse in the geriatric population
Source: Explor Res Clin Soc Pharm. 2025 Sep 30;20:100663. doi: 10.1016/j.rcsop.2025.100663 (PMC12547873; doi:10.1016/j.rcsop.2025.100663)
Supplement: Supplementary file 2 — Supplementary material 2 [file mmc2.docx]

Figure 1. The Distribution of Over-the-Counter (OTC) Medication Usage among Geriatric Patients (N=386)

**Table 1. Geriatric Patients' Knowledge and Awareness Regarding Non-Prescription Medication Use (N=386)**

| **Items** | **No** | **%** |
| --- | --- | --- |
| **Have you ever received any information or instructions about the safe use of non-prescription medications?** |  |  |
| Yes | 223 | 57.8% |
| No | 163 | 42.2% |
| **Do you know the dangers and complications of misusing non-prescription drugs?** |  |  |
| Yes | 234 | 60.6% |
| No | 152 | 39.4% |
| **Are you aware of interactions or interactions that may occur between over-the-counter and prescription medications?** |  |  |
| Yes | 134 | 34.7% |
| No | 168 | 43.5% |
| Not sure | 84 | 21.8% |
| **Did you know that some over-the-counter medications can negatively affect chronic health conditions?** |  |  |
| Yes | 208 | 53.9% |
| No | 105 | 27.2% |
| Not sure | 73 | 18.9% |
| **Are you aware of the potential side effects of the over-the-counter medications you are taking?** |  |  |
| Yes | 141 | 36.5% |
| No | 148 | 38.3% |
| Not sure | 97 | 25.1% |
| **Do you know the contraindications of over-the-counter medications?** |  |  |
| Yes | 169 | 43.8% |
| No | 217 | 56.2% |

**Table 2: Geriatric Patients' Practices, Perceptions, and Educational Needs Regarding Over-the-Counter Medication Use (N=386)**

|  | **No** | **%** |
| --- | --- | --- |
| **Have you taken your chronic conditions into account when taking over-the-counter medications?** |  |  |
| Never | 61 | 15.8% |
| Rarely | 51 | 13.2% |
| Sometimes | 158 | 40.9% |
| Always | 116 | 30.1% |
| **Do you read the instructions on the packages of non-prescription medications?** |  |  |
| Never | 79 | 20.5% |
| Rarely | 72 | 18.7% |
| Sometimes | 125 | 32.4% |
| Always | 110 | 28.5% |
| **Do you consult a pharmacist when purchasing non-prescription medications?** |  |  |
| Never | 31 | 8.0% |
| Rarely | 53 | 13.7% |
| Sometimes | 154 | 39.9% |
| Always | 148 | 38.3% |
| **Do you think healthcare professionals should ask about your use of over-the-counter medications?** |  |  |
| Yes | 331 | 85.8% |
| No | 55 | 14.2% |
| **Do you think there is a need for more awareness about the misuse of non-prescription drugs, especially among the elderly?** |  |  |
| Yes | 336 | 87.0% |
| No | 50 | 13.0% |
| **Would you like to receive educational materials on the safe use of non-prescription medications?** |  |  |
| Yes | 308 | 79.8% |
| No | 78 | 20.2% |

**Figure 2. Geriatric Patients' Suggested Methods for Over-the-Counter Medication Misuse Awareness Campaigns.**
